# Failure to rescue the trial: lessons from a randomised antibiotic treatment trial in the acute care setting

**DOI:** 10.1186/s13063-026-09722-3

**Published:** 2026-04-23

**Authors:** Sharon E. J. D. van den Eijnde, Eva L. Koekenbier, Paul D. van der Linden, Jan Jelrik Oosterheert, Marc J. M. Bonten, Cornelis H. van Werkhoven

**Affiliations:** 1 Julius Centre for Health Sciences and Primary Care, University Medical Centre Utrecht, Utrecht, the Netherlands; 2 Department of Pharmacy, Tergooi Medical Centre, Hilversum, the Netherlands; 3 Department of Internal Medicine, University Medical Centre Utrecht, Utrecht, the Netherlands; 4 ECRAID, European Clinical Research Alliance on Infectious Diseases, Utrecht, the Netherlands; 5 Department of Internal Medicine, Catharina Hospital, Eindhoven, the Netherlands; 6 Department of Acute Care, Department of Internal Medicine, Department of Clinical Pharmacy & Pharmacology, University Medical Centre Groningen, University of Groningen, Groningen, the Netherlands; 7 Department of Internal Medicine, Canisius Wilhelmina Hospital, Nijmegen, the Netherlands; 8 Department of Internal Medicine, Elkerliek Hospital, Helmond, the Netherlands; 9 Department of Internal Medicine, Meander Medical Centre, Amersfoort, the Netherlands; 10 Department of Internal Medicine, Medical Centre Leeuwarden, Leeuwarden, the Netherlands; 11 Department of Internal Medicine, Division of Infectious Diseases, Amsterdam University Medical Center, University of Amsterdam, Amsterdam, the Netherlands

**Keywords:** Clinical trial, Sepsis, Aminoglycoside, Barriers, Antibiotics

## Abstract

**Background:**

A cluster-randomised cross-over trial assessing whether cephalosporin monotherapy is non-inferior to cefuroxime plus short-course aminoglycoside combination therapy for empirical sepsis treatment was terminated for operational futility due to low enrolment and low protocol adherence. We evaluated barriers to enrolment and treatment compliance.

**Methods:**

Trial enrolment was between May 2022 and May 2023. For this mixed-methods study, we used questionnaires to identify barriers to enrolment and prescribing randomised treatment among local research teams (LRTs) and local clinical staff, which were obtained between January and March 2024. In addition, we investigated screening logs for non-enrolment reasons and determined patient characteristics associated with non-compliance. Clinicians being part of LRT received questionnaires for both roles, with their role clearly indicated per question.

**Results:**

In total, 65 questionnaires were completed: 23 by LRT members and 54 by clinical staff (including 12 LRT members). For patient enrolment, screening of inclusion and exclusion criteria was considered challenging in one hospital. According to screening logs, 52.2% of patients were excluded due to at least one exclusion criterion, and 15.6% of eligible patients declined consent for study participation. Primary barriers to prescribe combination therapy were concerns about potential side effects and limited perceived benefit compared to monotherapy. Secondary barriers included supervisors recommending deviations from study protocol. Keeping clinical staff informed and trained during the study was reported as relevant barrier for implementation by LRTs, but considered of low importance by clinical staff. No barriers were reported for monotherapy.

**Conclusions:**

In this study, the main barrier for patient enrolment was the higher than expected proportion of patients with exclusion criteria. The main barrier for treatment compliance was concerns about potential side effects of combination therapy. We recommend quantitative and qualitative pilot studies to identify barriers during the trial design phase, to optimise recruitment strategies and protocol adherence.

**Trial registration:**

European Union Clinical Trials Register, EUCTR2021-001840-83-NL. Registered on 7 July 2021, https://www.clinicaltrialsregister.eu/ctr-search/search?query=eudract_number:2021-001840-83

**Supplementary Information:**

The online version contains supplementary material available at 10.1186/s13063-026-09722-3.

## Background

In cluster-randomised cross-over (CRXO) trials, entire groups, such as wards, complete hospitals or cities, are randomised instead of individual patients [1]. This design is particularly well-suited when individual randomisation is infeasible, for instance when immediate intervention is needed [2]. Treatment of sepsis, a life-threatening condition characterised by organ dysfunction resulting from an abnormal and uncontrolled response of the host to infection, may serve as an example [3]. For sepsis, antibiotic treatment should be initiated as soon as possible upon arrival at the emergency department (ED) [4]. In the Netherlands, the most widely used empirical antibiotic regimens for sepsis are ceftriaxone monotherapy or combination therapy of cefuroxime and a short-course of aminoglycosides. Both regimens provide adequate coverage against the most likely causative pathogens and are recommended in the Dutch sepsis guideline [5]. Adding aminoglycosides extends pathogen coverage and presumably enhances bacterial killing, but may also cause nephrotoxicity and ototoxicity [6, 7].

The *Short-course AminoGlycosides as Adjunctive treatment in adults with sepsis*trial (SAGA) was a CRXO designed to determine if cephalosporin monotherapy is non-inferior to a combination of cephalosporin with short-course aminoglycoside in reducing 30-day mortality [8]. The cross-over design enabled each hospital to act as its own control, reducing the effects of between-hospital variability. As current Dutch law on research involving human subjects does not permit opt-out procedures for data collection in cluster-randomised studies, a deferred consent procedure was implemented, even though both antibiotic regimens are considered standard care practice and widely used. The trial encountered two major challenges: low inclusion rates and high non-compliance with the randomised antibiotic treatment in the combination therapy group. In the initial sample size calculation, a 90% adherence to the randomised treatment was assumed, but only 54% of patients received the intended treatment in the combination therapy arm, compared to 91% in the monotherapy arm. Several mitigation strategies failed to improve compliance, as did interventions to increase enrolment, including adding hospitals to the study. As a result, the trial was prematurely terminated due to non-feasibility, before cross-over had occurred.

Approximately one-third of all randomised trials are prematurely discontinued, and such trials have a higher odds of remaining unreported [9]. A comprehensive assessment of trial failure is therefore important to inform future trial design, reduce the suboptimal use of research resources and promote transparent research. This study evaluated the reasons for trial failure by identifying barriers to enrolment and treatment compliance.

## Methods

### Trial design

The SAGA trial was conducted in six secondary and three tertiary care hospitals in the Netherlands between May 2022 and May 2023 [8]. Four hospitals were randomised to cephalosporin monotherapy (cefuroxime or ceftriaxone) and five to combination therapy of cefuroxime and short-course aminoglycoside (gentamicin). The randomised regimen was adopted into local antibiotic treatment guidelines as the preferred empirical therapy for patients with community-acquired sepsis of unknown, suspected urinary tract or abdominal origin. This approach was taken to ensure and communicate that each hospital’s antibiotic committee and principal investigator (PI) endorsed the equipoise between the two treatment strategies. Local research team (LRT) members received study training prior to study initiation. PIs were responsible for training and informing local clinical staff involved in sepsis treatment, while central coordinating investigators were available to train the clinical staff upon PI request. Pocket cards and posters summarising the study were distributed to enhance treatment compliance.

All patients admitted to the emergency department were screened for eligibility; the number screened was not recorded. Among those who met the inclusion criteria, screening for exclusion criteria was systematically recorded [8]. Due to the acute care setting, a deferred consent procedure was used, with consent to be obtained within 72 h of hospitalisation. For incapacitated patients, consent was obtained from the legal representative. LRT members regularly assessed the patient’s capacity to obtain consent. If incapacity persisted beyond 30 days, both patient and legal representative were notified in writing that hospitalisation and 30-day survival data was collected for the study. Adherence to randomised treatment was monitored monthly by the central coordinating team, starting 6 months after trial initiation, and reported to PIs to enhance compliance. More details on the trial design are provided elsewhere [8].

### Study design and data collection

This mixed-method study included an online questionnaire emailed to the nine PIs of the SAGA trial, who were requested to distribute the questionnaire to LRT members and local clinical staff, including physicians and nurses involved in sepsis treatment. Distribution was not recorded. The estimated response rate was based on the number of local medical staff members at the time of the evaluation. The questionnaire was developed by the central coordinating team and addressed four themes: (1) training and preparation, (2) study procedures other than themes 3 and 4, (3) deferred consent procedure and (4) randomised empirical treatment prescribing. Questions regarding all four themes were directed to the LRTs, while the clinical staff were only asked questions related to theme 4. Some LRT members also worked as clinicians. Consequently, individuals working in both roles received questions directed to LRT members as well as to clinical staff. Each question specified the role for which the question was intended. For example, for the LRT role, they were asked what they, as LRT members, perceived as the most important barrier among clinicians for prescribing combination therapy, whereas for the clinician role, they were asked about the most important barrier for themselves. The complete questionnaire consisted of 33 questions with a five-point Likert scale and 11 open-ended questions (see Supplement S1 and S2). The open-ended questions were optional and allowed participants to elaborate on their responses or add additional comments. Additionally, barriers to prescribing the randomised treatment were ranked from most important (= 1) to least important (= 6 or 7, depending on the number of barriers). Data were collected using Google Forms between 23 January and 7 March 2024, with a reminder sent to the PIs on 13 February 2024.

For further identification of barriers to enrolment, screening logs from the SAGA trial were reviewed for exclusion criteria and non-consent. To further investigate determinants of non-compliance, associations between patient characteristics and non-compliance were analysed in the combination therapy group. This was not done for the monotherapy group, as the adherence rate exceeded 90%.

Non-compliance to the randomised treatment increases the required sample size to obtained sufficient power for a pursued study outcome. One could argue to add exclusion criteria related to physician hesitancy to comply with the randomised study treatment. This could indeed reduce non-compliance, but would also reduce the number of eligible patients for the study pool, thereby increasing the required trial duration if the number of study sites is fixed. To better inform future trial designs, the effect of implementing stricter study criteria on relative trial efficiency was assessed. For this, we calculated effective sample size of the restricted group (“compliance effect”) and the relative change in eligible patients (“eligibility effect”). The compliance effect reflected the relative change in sample size due to differences in non-compliance before and after applying stricter study criteria. The relative effect of non-compliance on the required sample size was calculated as 1/(1 − e1 − e2)^2, where e1 and e2 are the non-compliance rates in the two study arms [10]. The eligibility effect was defined as the proportion of patients remaining eligible under the stricter criteria. Relative trial efficiency can be calculated as the product of compliance and eligibility effect and represents the relative decrease in required trial duration given a fixed number of sites. See Supplement S3 for further elaboration and example calculations.

### Statistical analysis

Means and standard deviations (SD) were calculated for the ranking questions. Answers to open-ended questions were scrutinised (by author SvdE) and presented as quotes. No thematic analysis could be performed due to the design of the questionnaire. Results were aggregated by randomisation arm. To analyse factors associated with non-compliance, multivariable logistic regression was conducted, using non-compliance as the dependent variable and baseline characteristics as the independent variables. Results were reported as odds ratios (OR). Additionally, both individual and joint population attributable fractions (PAF), i.e. the proportion of outcomes that could be avoided if the risk factor was fully eliminated (i.e. if the factor was no longer a reason for physicians to be non-compliant), as well as the effect of stricter study criteria on trial efficiency (i.e. if the factor would be added as an exclusion criterion), were calculated for each factor with a *p* value < 0.2 in the multivariable logistic regression analysis. The non-explained fraction was determined as one minus the joint PAF. Reasons for non-enrolment were descriptively analysed using frequencies. Analyses were performed using R version 4.4.0.

## Results

### Respondent characteristics

LRT members completed 23 questionnaires and local clinical staff completed 54 questionnaires, of whom 42 (78%) were not LRT members (Table 1). An estimated total of 742 physicians were involved in sepsis treatment during the study period, of whom 359 were residents and 383 medical specialists. Only one nurse completed the questionnaire. It was decided post hoc to review the nurse’s response separately from the physicians and exclude it from the summary statistics.

**Table 1:** Baseline characteristics

**Local research teams**	**Combination therapy (*N*** **= 12)**	**Monotherapy (*N*** **= 11)**	**All participants (*N*** **= 23)**
Principal investigator	4 (33.3%)	4 (36.4%)	8 (34.8%)
(Medical) researcher	3 (25.0%)	2 (18.2%)	5 (21.7%)
Other research staff^a^	5 (41.7%)	5 (45.5%)	10 (43.5%)
**Local clinical staff**	**Combination therapy (*N*** **= 36)**	**Monotherapy (*N*** **= 18)**	**All participants (*N*** **= 54)**
Medical specialist	23 (63.9%)	15 (83.3%)	38 (70.4%)
Resident	12 (33.3%)	3 (16.7%)	15 (27.8%)
Nurse	1 (2.8%)	0 (0%)	1 (1.9%)

^a^Including research associates (*n* = 3), research nurses (*n* = 2), data managers (*n* = 1) and other (*n* = 4)

### Training and preparation

In the monotherapy arm, 90% of LRT members reported to be adequately trained prior to the study, compared to 78% in the combination therapy arm. Furthermore, 89% of LRT members in the monotherapy and 70% in the combination therapy arm reported receiving sufficient training opportunities. However, 30% of LRT members in the combination therapy reported that they should have provided more training to the clinical staff, supported by the following quote: “… This [training the physicians and nurses, SvdE] might have helped to directly mitigate any fear/resistance from previous experiences”. For the other questionnaire items, only minor differences were observed between the two groups (Fig. S4).

### Study procedures

In the combination therapy arm, 38% of the LRT members perceived that physicians did not see added value in participating in the study, compared to 10% in the monotherapy arm. Overall, 70% of combination therapy and 90% of monotherapy LRT members considered the clinical staff to be sufficiently informed to carry out the procedures. However, training of clinical staff was perceived as difficult: “Training and maintaining a broad group of clinical staff informed with a continuous flow of new people [personnel, SvdE] is challenging”. One respondent remarked that “… There were some inclusion and exclusion criteria that hindered the implementation of the study. …”, without specifying which criteria. It remains uncertain whether this affected patient enrolment. For the remaining questionnaire items, only small between-group differences were observed (Fig. S5). In total, 1169 patients met the inclusion criteria; however, 610 (52.2%) met at least one exclusion criterion, leaving 559 patients eligible for study participation: 311/656 (47.4%) in the hospitals allocated to combination therapy and 248/513 (48.3%) in those allocated to monotherapy [8]. The most common exclusion criterion in both arms was the presence of an indwelling urinary catheter or intermittent catheterisation prior to sepsis onset (*n* = 265), followed by a history of pre-existing renal failure (eGFR < 30 ml/min) or kidney transplantation (*n* = 121).

### Deferred consent procedure

Most respondents considered the consent procedure of added value for both patients (74%) and study (76%); specification of the added value was not requested nor spontaneously provided. Two respondents did not consider the consent procedure feasible to implement within their study team. An alternative approach was suggested: “… In my opinion, the study would have been better conducted using an opt-out (non-objection) approach…”. The results for the remaining questionnaire items are presented in Fig. S6. In the trial, 48 of 311 (15.4%) eligible patients declined consent in the combination therapy arm and 39 of 248 (15.7%) patients in the monotherapy arm.

### Prescribing of randomised empirical treatment

According to three (33%) LRT members, clinical staff (non-LRT members) in hospitals randomised to combination therapy were reluctant to adhere to protocol and were concerned it might result in inferior treatment for patients (Fig. S7). Four (44%) LRT members reported that fear of potential side effects hindered prescribing of combination therapy, with eight (67%) identifying this as the most or second most important barrier. These findings were consistent with responses from physicians, of whom 17 (49%) indicated that fear of potential side effects hindered protocol adherence, and 19 (54%) identified it as the most or second most important barrier (Table 2 and Fig. S8). Prior experiences made five physicians hesitant to administer combination therapy (15%). Additionally, seven residents (58%) reported supervisors recommending deviation from combination therapy, compared to 19% of medical specialists (Figs. S8, S8a and S8b). Thirteen (37%) identified this as the most or second most important barrier (Table 2). Challenges with applying inclusion and exclusion criteria at the ED and a limited perceived benefit of combination therapy were also mentioned as barriers by LRT members. One LRT member noted, “The need to add gentamicin in a population that generally has a favourable outcome with standard therapy resulted in the risk/benefit ratio being viewed as marginal in advance”. Physicians mentioned instances where antibiotics were administered before consultation, lack of information about the study, and a preference for autonomy in deciding when to use aminoglycosides. One respondent concluded that “… the ‘fear’ of gentamicin (nephrotoxicity and ototoxicity) and hassle (therapeutic drug monitoring) negatively impacted protocol adherence”. The nurse identified unfamiliarity with the protocol as the most important barrier, followed by physicians recommending deviation.

**Table 2:** Ranking of barriers to prescribing randomised empirical treatment

**Local research teams**	**Combination therapy (*n *= 12)**		**Monotherapy (*n *= 11)**	
	**Mean**^**a**^	**Standard deviation (SD)**	**Mean**^**a**^	**SD**
Unfamiliarity with the local antibiotic protocol/the randomised treatment	3.75	2.14	2.91	2.07
Insufficiently convinced of the higher effectiveness of the randomised treatment	2.67	1.72	3.18	1.54
Lower effectiveness of the randomised treatment	3.67	0.98	2.36	1.21
Frequency of administration	3.58	1.00	3.73	1.19
Concerns for potential side effects	2.75	1.76	4.36	1.29
Other	4.58	1.93	4.45	2.07
**Physicians**	**Combination therapy (*n *= 35)**		**Monotherapy (*n *= 18)**	
	**Mean**^**b**^	**SD**	**Mean**^**b**^	**SD**
Unfamiliarity with the local antibiotic protocol/the randomised treatment	4.20	2.36	4.50	2.50
Insufficiently convinced of the higher effectiveness of the randomised treatment	4.46	1.70	4.33	1.97
Lower effectiveness of the randomised treatment	4.54	1.44	3.83	1.65
Supervisor recommending deviation	3.66	1.98	3.00	1.75
Frequency of administration	3.94	1.61	4.17	1.58
Concerns for potential side effects	2.83	1.95	4.22	1.66
Other	4.37	2.39	3.94	2.62

^a^Range between 1 and 6; low values indicate high perceived importance of the barrier

^b^Range between 1 and 7; low values indicate high perceived importance of the barrier

**Fig. 1:**
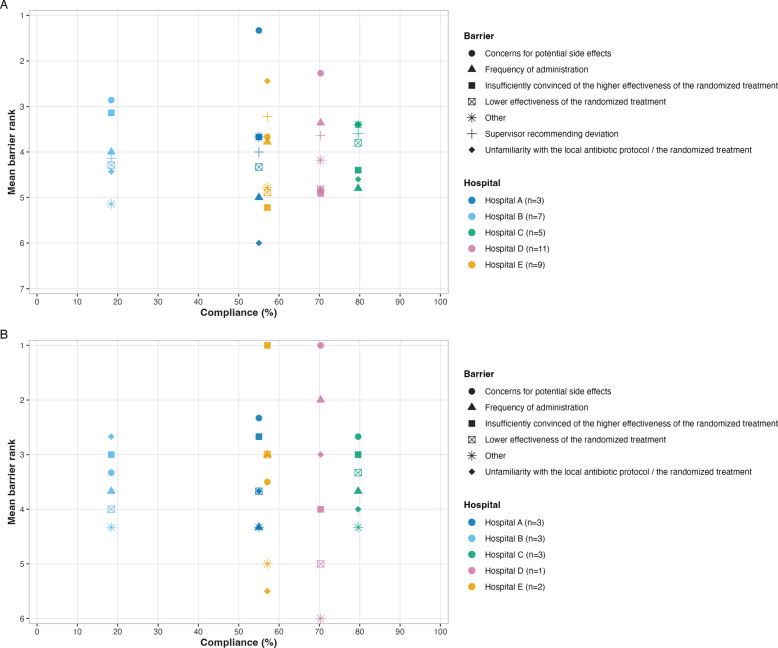
Mean barriers per hospital randomised to combination therapy. **A** Physicians. Symbols represent mean barrier ranks on a scale from 1 (most important) to 7 (least important) for barriers related to prescribing combination therapy. Hospital letters correspond to those used in Table 3. **B** Local research teams. Symbols represent mean barrier ranks on a scale from 1 (most important) to 6 (least important) for barriers related to prescribing combination therapy. Hospital letters correspond to those used in Table 3

Based on mean barrier rankings per hospital, concerns for potential side effects was identified as the most important barrier to prescribing combination therapy among physicians in all hospitals except hospital E (Fig. 1A). In hospital E, which had mid-range compliance, unfamiliarity with the local antibiotic protocol and the randomised treatment was ranked as the most important barrier by physicians, whereas LRTs ranked this as the least important barrier (Fig. 1B). Two hospitals (A and B) ranked supervisor recommendations to deviate from protocol as a less important barrier than the other hospitals. These hospitals had the lowest compliance, although hospital A is close in compliance to hospital E.

For ceftriaxone monotherapy, five LRT members (45%) ranked the perceived lower effectiveness as the first or second barrier, whereas eight (44%) physicians did so for supervisors recommending deviations from protocol (Table 2). Nevertheless, both groups frequently noted that they did not recognise any true barriers for monotherapy. However, one LRT member indicated that, “Sometimes it [current empirical treatment protocol, SvdE] was forgotten”, and two respondents identified insufficient information as a barrier for implementation.

**Fig. 2:**
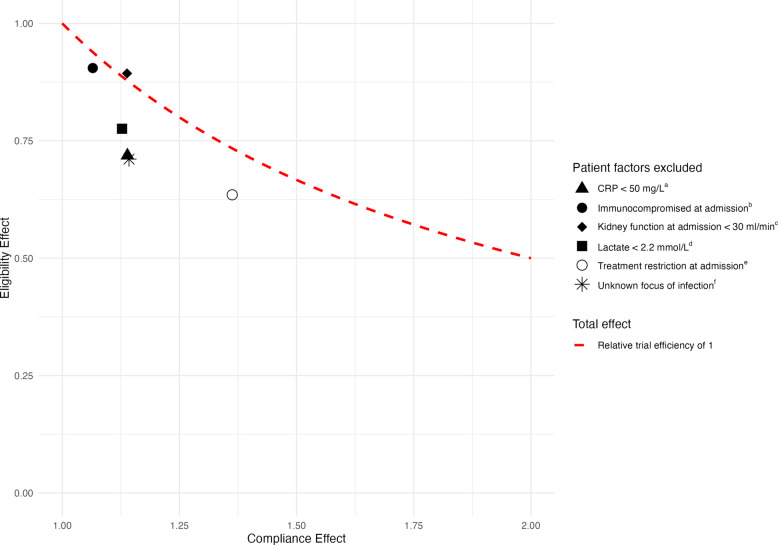
Effect of stricter study criteria on compliance, eligibility and relative trial efficiency. Effective sample size (ESS) of the restricted group (“compliance effect”) and relative change in eligible patients (“eligibility effect”). An ESS of 1.25 implies that the sample size can be 80% (1/1.25) of the original sample size. Relative trial efficiency was calculated as the product of these effects. A value > 1 (right upper area) indicates trial duration would have been shorter with the same participating hospitals. The expected change in trial duration if the same sites were participating is given by [1/relative trial efficiency]. Possible differences in primary outcome incidence and acceptable non-inferiority margin were not taken into account. Further explanation and calculated examples are provided in Supplement S3. ^a^Excluding patients with CRP levels < 50 mg/L reduced non-compliance from 46.4% to 43.4% and decreased the number of eligible patients from 263 to 189, resulting in an 18.1% reduction in the ESS. ^b^Excluding immunocompromised patients reduced non-compliance from 46.4% to 45.0% and decreased the number of eligible patients from 263 to 238, resulting in a 3.6% reduction in the ESS. ^c^Excluding patients with a kidney function at admission < 30 ml/min reduced non-compliance from 46.4% to 43.4% and decreased the number of eligible patients from 263 to 235, resulting in an 1.7% increase in the ESS. ^d^Excluding patients with lactate levels < 2.2 mmol/L reduced non-compliance from 46.4% to 43.6% and decreased the number of eligible patients from 263 to 204, resulting in a 12.5% reduction in the ESS. ^e^Excluding patients with a treatment restriction reduced non-compliance from 46.4% to 38.9% and decreased the number of eligible patients from 263 to 167, resulting in a 13.5% reduction in the ESS. ^f^Excluding patients with an unknown focus of infection reduced non-compliance from 46.4% to 43.3% and decreased the number of eligible patients from 263 to 187, resulting in an 18.8% reduction in the ESS

In the combination therapy arm, non-compliance was significantly associated with the hospital of admission, unknown primary focus of infection, CRP ≥ 50 mg/L, presence of a treatment restriction and kidney function of <30 ml/min at admission (Table 3). When excluding hospital B (with the lowest compliance), hospital of admission, unknown primary focus of infection and kidney function < 30 ml/min at admission remained significantly associated. Kidney function at admission had a PAF of 18.0% (95% CI: 6.4–28.3) (Table 3), and together, clinical factors explained 59.7% (95% CI: 35.1–76.6) of non-compliance. When combined with variation between hospitals, these factors accounted for 94.7% (95% CI: 81.6–98.4). Excluding patients with a kidney function < 30 ml/min at admission resulted in an 2% increase in trial efficiency, whereas the exclusion of other patient factors led to a reduced trial efficiency (Fig. 2).

**Table 3:** Risk factors associated with non-compliance

Variables	Antibiotic therapy received^a^		Effect estimate	
	Combination therapy (compliant) *N* = 141	No combination therapy (non-compliant) *N* = 122	Odds ratio (95% CI)	Population attributable fraction (95% CI)
Hospital^b^				0.62 (0.40–0.76)^d^
Hospital A	11 (7.8%)	9 (7.4%)	Ref	–
Hospital B	14 (9.9%)	62 (50.8%)	8.33 (2.51–29.52)	–
Hospital C	43 (30.5%)	11 (9.0%)	0.25 (0.06–0.91)	–
Hospital D	45 (31.9%)	19 (15.6%)	0.65 (0.19–2.27)	–
Hospital E	28 (19.9%)	21 (17.2%)	1.02 (0.28–3.68)	–
Age				NA
<71 years	76 (53.9%)	49 (40.2%)	Ref	–
≥71 years	65 (46.1%)	73 (59.8%)	1.34 (0.63–2.90)	–
Sex				NA
Female	64 (45.4%)	48 (39.3%)	Ref	–
Male	77 (54.6%)	74 (60.7%)	1.04 (0.54–2.00)	–
NEWS				NA
≥5 and <7	50 (35.5%)	45 (36.9%)	Ref	–
≥7	91 (64.5%)	77 (63.1%)	0.79 (0.38–1.61)	–
Focus of infection^b^				0.12 (0.002–0.22)^e^
Urinary tract	72 (51.1%)	58 (47.5%)	Ref	–
Abdominal origin	34 (24.1%)	23 (18.9%)	1.51 (0.62–3.70)	–
Unknown	35 (24.8%)	41 (33.6%)	2.66 (1.24–5.86)	–
CRP^b^				0.09 (0.02–0.16)^f^
<50 mg/L	34 (24.1%)	40 (32.8%)	Ref	–
≥50 mg/L	107 (75.9%)	82 (67.2%)	0.36 (0.17–0.76)	–
Leukocytes				NA
<4 × 10^9/L	9 (6.4%)	1 (0.8%)	0.24 (0.01–1.93)	–
≥4 and <11 × 10^9/L	50 (35.5%)	46 (37.7%)	Ref	–
≥11 × 10^9/L	82 (58.2%)	75 (61.5%)	1.28 (0.66–2.54)	–
Lactate^b^				0.09 (−0.09–0.26)^g^
<2.2 mmol/L	26 (18.4%)	33 (27.0%)	Ref	–
≥2.2 mmol/L	47 (33.3%)	40 (32.8%)	0.54 (0.21–1.35)	–
No lactate measured	68 (48.2%)	49 (40.2%)	0.59 (0.24–1.40)	–
Kidney function at admission^b^				0.18 (0.06–0.28)^h^
<30 ml/min	8 (5.7%)	20 (16.4%)	6.17 (1.73–23.72)	–
≥30 and <60 ml/min	42 (29.8%)	44 (36.1%)	2.00 (0.93–4.41)	–
≥60 ml/min	77 (54.6%)	53 (43.4%)	Ref	–
No kidney function measured	14 (9.9%)	5 (4.1%)	1.02 (0.25–3.70)	–
Treatment restriction at admission^b^	39 (27.7%)	57 (46.7%)	2.34 (1.12–5.00)	0.14 (0.05–0.22)^i^
Immunocompromised at admission^b^	10 (7.1%)	15 (12.3%)	2.75 (0.83–9.55)	0.03 (−0.007–0.06)^j^
Kidney function in the prior 3 months				NA
≥30 and <60 ml/min	11 (7.8%)	25 (20.5%)	1.31 (0.41–4.24)	–
≥60 ml/min	60 (42.6%)	39 (32.0%)	Ref	–
No kidney function measured	70 (49.6%)	58 (47.5%)	0.95 (0.45–2.01)	–
History of renal disease	5 (3.5%)	12 (9.8%)	2.26 (0.53–10.17)	NA
Prior history of disease^c^	104 (73.8%)	104 (85.2%)	1.25 (0.52–3.09)	NA

*CI* Confidence interval, *NEWS* National early warning score, *NA* Not applicable

^a^Only patients from hospitals randomised to the combination therapy were included

^b^Variable with a *p* value < 0.2 in the multivariable logistic regression

^c^History of rheumatic disease, cardiovascular disease, peripheral vascular disease, diabetes mellitus, hypertension, chronic pulmonary disease, COPD, cerebrovascular disease, dementia, hemiplegia or paraplegia, peptic ulcer disease, moderate to severe liver disease, malignancy or HIV/AIDs

^d^Hospitals A, B, D and E compared to hospital C

^e^Gastrointestinal tract and unknown focus of infection compared to urinary tract

^f^CRP < 50 mg/L compared to CRP ≥ 50 mg/L

^g^Lactate < 2.2 mmol/L and no measured lactate compared to lactate ≥ 2.2 mmol/L

^h^Kidney function < 30 ml/min, ≥30 and <60 ml/min, and no measured kidney function compared to kidney function ≥ 60 ml/min

^i^Treatment restriction compared to no treatment restriction

^j^Immunocompromised compared to not being immunocompromised

## Discussion

In this study, we identified key barriers to enrolment and compliance with randomised empirical antibiotic treatment in a CRXO trial comparing cephalosporin monotherapy to cephalosporin combined with short-course aminoglycoside in sepsis patients. The most important barriers to adhering to the randomised treatment included concerns about potential side effects and a lack of perceived benefit in hospitals randomised to combination therapy, while no substantial barriers were reported for monotherapy. In both arms, recommendations from supervisors to deviate from protocol and challenges in ensuring that clinical staff remained adequately informed and trained were reported as relevant barriers. For enrolment, screening of inclusion and exclusion criteria was considered challenging in one hospital. Overall, 52.2% of patients were excluded due to at least one exclusion criterion, and 15.6% of eligible patients declined consent to participate in the study. The deferred consent procedure was generally regarded as adding value for both the patients and the study, although some considered this as challenging for LRTs to implement. The concerns about side effects were confirmed in the quantitative analysis, which identified kidney function at admission as an important explanatory variable with large differences between hospitals.

Individual-based randomisation is a challenge when treatment must start immediately, as holds for antibiotic therapy in patients with sepsis. In many trials using such a design, randomised treatment could only start after empiric treatment had already been given. For such interventions, a cluster randomised design, in which the different interventions are temporarily considered the standard of care, would provide a better estimate of the treatment effects as the randomised treatment would also be the initial treatment. Yet, in such a design, non-adherence to the randomised treatment is a major challenge and is often underreported [11]. Proposed strategies to expedite cluster randomised trial adoption include appointing gatekeepers to inform staff and conduct educational meetings [12]. In the SAGA trial, local PIs were responsible for informing and training local clinical staff. Although all local PIs supported the study’s relevance and design, some hospitals encountered unanticipated challenges in securing protocol adherence, resulting in considerable variability in compliance. By applying strict inclusion and exclusion criteria, variation in case-mix between hospitals was minimised, and multivariable analysis confirmed that between-hospital variation in patient characteristics did not explain the observed differences in compliance between hospitals. To identify factors associated with non-compliance at the individual level, this study primarily focused on odds ratios. However, a large odds ratio for a low-prevalence factor may contribute less to overall non-compliance than a smaller odds ratio for a high-prevalence factor. Therefore, PAFs were calculated to complement odds ratios by providing a population-level perspective on non-compliance. If compliance would reach the level of the best performing hospital, taking into account patient mix, the non-compliance would have decreased by 62%. Despite efforts to motivate staff, it proved difficult to maintain their engagement and overcome their hesitancy to prescribe combination therapy. High personnel turnover further hindered consistent training and sustained involvement, particularly given the high proportion of patients who met the exclusion criteria. In each hospital, there was one PI, usually an ID physician. Yet, many other medical specialities are also involved in sepsis treatment and could affect adherence rates by recommending deviations from local antibiotic guidelines and, consequently, from study protocol. Their perspectives on the trial could have been more thoroughly determined before study start, as there appeared to be a gap in perceived equipoise between the PIs, antibiotic committees and involved clinical staff. Restricting the eligible population by excluding patients at higher risk of non-compliance, e.g. with an eGFR < 30 ml/min, would not have reduced the required trial duration.

Applying inclusion and exclusion criteria in the ED was perceived as challenging. Based on subsequent discussions with LRT members, we hypothesised that physicians applied the criteria before initiating empirical treatment, possibly to avoid administering aminoglycosides. To improve adherence rates in future randomised studies in acute care, using electronic health record systems for screening and randomisation may be a viable solution, as used in a recent study [13]. In that study, physicians had to directly motivate a deviation from the study protocol, which potentially reduces non-compliance.

Based on our survey and screening log data, we concur that the consent procedure has not substantially contributed to low recruitment. Instead, the lower than anticipated number of eligible patients was the primary reason for slow recruitment. To address this, additional hospitals were approached. Unfortunately, some had recently revised their local antibiotic policy from combination therapy to monotherapy, and a reversal of their treatment protocols in the context of this study was perceived as potentially confusing and disruptive. Other hospitals declined due to a substantial increase in workload following the COVID-19 pandemic. Finally, some hospitals declined participation due to growing reluctance among their physicians to use aminoglycosides in sepsis patients. This was echoed in our findings, where the perceived limited benefit of combination therapy together with fear of side effects was identified as a major barrier to its implementation. It remained unclear whether opinions had changed, and if so, why they had evolved in this direction, despite the absence of new supporting data.

Our findings can be further interpreted using the Consolidated Framework for Implementation Research (CFIR), which provides insight into understanding barriers to implementation effectiveness [14]. Barriers related to concerns about potential side effects and a lack of perceived benefit align with the Innovation domain, particularly the constructs of relative advantage and evidence base, suggesting that physicians’ perceptions of the antibiotics’ clinical value and safety were key to prescribing behaviour. The reported complexity of the enrolment procedure further reflects perceived innovation complexity and may have contributed to reduced enrolment across hospitals. Supervisor recommendations to deviate from study protocol corresponds to the Individuals domain, specifically the influence of leaders, highlighting the role of leadership in clinical decision-making. In addition, challenges in maintaining adequate staff training align with the Inner Setting domain, particularly the constructs of communication and access to knowledge and information. Taken together, these findings indicate that barriers emerged across multiple domains, underscoring the importance of addressing these different contextual factors when designing and implementing trials in acute care settings.

The study has several limitations. First, because questionnaires were distributed through PIs and detailed records of questionnaire distribution were not recorded, we were unable to calculate exact response rates. Although PIs were instructed to forward questionnaires to all physicians and nurses involved, some sent them exclusively to physicians, resulting in underrepresentation of nurses. Second, the high turnover of residents and distribution of questionnaires 10 months after the discontinuation of the SAGA trial may have resulted in a low representation of residents among respondents. Indeed, the majority of respondents were medical specialists, and variations in clinical experience could have influenced their responses. Specialists are likely to prioritise adverse effects based on prior experiences, whereas residents may be more influenced by the guidance of their supervisors. Third, the survey results are prone to information bias, particularly in the combination therapy arm. Their responses may have been influenced by a desire to justify the high non-compliance rates observed in their hospitals. Fourth, selection bias arising from variability in questionnaire response cannot be excluded, as individuals with stronger opinions may have been more likely to participate. Fifth, although the per-hospital rankings may shed some light on potential organisational and cultural factors contributing to inter-hospital variability, these were not assessed in depth in the current study and remain an important area for future investigation. Finally, our findings are based on experiences from a single trial, which may limit generalisability. Therefore, future research is needed to validate this across other trials.

## Conclusions

The main barrier to adhere to the randomised empirical treatment protocol was concern regarding the potential side effects of combination therapy. Secondary barriers included supervisors’ recommendations to deviate from the protocol and insufficient training among the clinical staff involved in sepsis treatment. To mitigate these risks in future trials, targeted educational interventions addressing the safety and rationale of the study should be conducted across all relevant medical departments to promote consensus regarding the study protocol. In addition, regular educational sessions facilitated by the central coordinating team are recommended to maintain engagement, improve compliance and address concerns throughout the study period. Implementing a CRXO study in an acute care setting requires clear inclusion and exclusion criteria that can be feasibly applied in the ED. A quantitative and qualitative pilot study could provide valuable insights into patient eligibility, enrolment pace and clinical staff willingness to participate and adhere to the randomised treatment, allowing early identification of potential challenges and timely adjustments to recruitment strategies or study design. Further research is needed to confirm the generalisability of our study to other trials in the acute care setting.

## Supplementary Information

Supplementary Material 1.

Supplementary Material 2.

## Data Availability

The questionnaire dataset is available from the corresponding author on reasonable request. The dataset used to analyse the determinants of non-compliance is available in the DataverseNL repository, https://doi.org/10.34894/NKVSYJ.

## Abbreviations

CRXO: Cluster-randomised cross-over
ED: Emergency department
LRT: Local research team
OR: Odds ratio
PAF: Population attributable fraction
PI: Principal investigator
SAGA: Short-course AminoGlycosides as Adjunctive treatment in adults with sepsis
SD: Standard deviation
