# Supplementary material for: Failure to rescue the trial: lessons from a randomised antibiotic treatment trial in the acute care setting
Source: Trials. 2026 Apr 23;27:418. doi: 10.1186/s13063-026-09722-3 (PMC13237994; doi:10.1186/s13063-026-09722-3)
Supplement: Supplementary file 1 — Supplementary Material 1. [file 13063_2026_9722_MOESM1_ESM.docx]

Supplement to:

Failure to rescue the trial: lessons from an antibiotic treatment trial in the acute care setting

This appendix has been provided by the authors to give readers additional information about their work.

**Table of Contents**

[S1. Translation of the questionnaire – Local research teams 2](#_Toc226118354)

[S2. Translation of the questionnaire – Clinical staff 5](#_Toc226118355)

[S3. Explanation and practical example of relative trial efficiency calculation 6](#_Toc226118356)

[S4. Results of training and preparation 8](#_Toc226118357)

[S5. Results of study procedures 9](#_Toc226118358)

[S6. Results of deferred consent procedure 10](#_Toc226118359)

[S7. Results of prescribing randomized empirical treatment – Local research teams 11](#_Toc226118360)

[S8. Results of prescribing randomized empirical treatment – Physicians 12](#_Toc226118361)

## S1. Translation of the questionnaire – Local research teams

**Theme 1.** Training/preparation

| Dutch | English |
| --- | --- |
| Voorafgaand aan de studie ben ik voldoende getraind door het centrale onderzoeksteam. | Prior to the study, I was adequately trained by the central research team. |
| Er zijn mij voldoende trainingsmogelijkheden aangeboden door het centrale onderzoeksteam. | I have been offered sufficient training opportunities by the central research team. |
| De training door het centrale onderzoeksteam omvatte inhoudelijk wat ik moest weten om de studie goed uit te kunnen voeren. | The training provided by the central research team covered the content I needed to know to properly conduct the study. |
| Het onderzoeksteams gaf duidelijk antwoord op vragen tijdens de training. | The research team clearly answered questions during the training. |
| Het onderzoeksteam was goed bereikbaar voor vragen tijdens de voorbereidingen. | The research team was readily available for questions during the preparations. |
| Het ziekenhuispersoneel (bv. artsen en verpleegkundigen) had meer getraind moeten worden door het lokale onderzoeksteam | Clinical staff (e.g., phycicians and nurses) should have been trained more by the local research team. |

**Theme 2.** Study procedures

| Dutch | English |
| --- | --- |
| Het screenen op inclusiecriteria van patiënten was goed uit te voeren in mijn ziekenhuis. | Screening for patient inclusion criteria was easily conducted in my hospital. |
| Artsen zagen toegevoegde waarde in deelname aan de studie. | Physicians saw added value in participating in the study. |
| Verpleegkundige zagen toegevoegde waarde in deelname aan de studie. | Nurses saw added value in participating in the study. |
| Het ziekenhuispersoneel (bv. artsen en verpleegkundigen) was voldoende op de hoogte van de studie om de studiehandelingen goed uit te kunnen voeren. | The clinical staff (e.g., physicians and nurses) were sufficiently informed about the study to properly carry out the study procedures. |
| Het onderzoeksteam was goed bereikbaar voor vragen tijdens de studie. | The research team was readily available for questions during the study. |

**Theme 3.** Deferred consent procedure

| Dutch | English |
| --- | --- |
| De uitgestelde toestemmingsprocedure (= deferred consent procedure) zoals bedacht door het centrale team, zat goed in elkaar. | The deferred consent procedure, as conceptualized by the central team, was well-designed. |
| De deferred consent procedure was in de praktijk voor het studieteam goed uit te voeren. | The deferred consent procedure was feasible to implement for the study team. |
| De deferred consent procedure zorgde ervoor dat het studieteam de patiënt/wettelijk vertegenwoordiger op een geschikter moment kon benaderen. | The deferred consent procedure allowed the study team to approach the patient/legal representative at a more convenient time. |
| De tijdlijn om de patiënt/wettelijk vertegenwoordiger te benaderen (binnen 72 uur) was voor het studieteam goed haalbaar. | The timeline for approaching the patient/legal representative (within 72 hours) was feasible for the study team. |
| Doordat we de patiënt/wettelijk vertegenwoordiger op een later moment konden benaderen (in plaats van direct bij opname op de SEH) werden deelnemers beter geïnformeerd over de studie. | Because we were able to approach the patient/legal representative at a later time (rather than immediately upon admission to the emergency department), participants were better informed about the study. |
| De extra tijdsinvestering die nodig was voor de deferred consent procedure is in verhouding met de extra meerwaarde die het heeft. | The additional time investment required for the deferred consent procedure was proportional to the added value it provided. |
| Het was voor de patiënt/wettelijk vertegenwoordiger duidelijk waarom de deferred consent procedure plaatsvond. | It was clear to the patient/legal representative why the deferred consent procedure took place. |
| De deferred consent procedure heeft eraan bijgedragen dat patiënten eerder ja hebben gezegd dan als er direct bij presentatie op de spoedeisende hulp toestemming werd gevraagd. | The deferred consent procedure contributed to patients being more inclined to consent than if consent was sought immediately upon presentation at the emergency department. |
| De deferred consent procedure heeft eraan bijgedragen dat wettelijk vertegenwoordigers eerder ja hebben gezegd dan als er direct bij presentatie op de spoedeisende hulp toestemming werd gevraagd. | The deferred consent procedure contributed to legal representatives being more inclined to consent than if consent was sought immediately upon presentation at the emergency department. |
| Alles samengenomen vind ik de deferred consent procedure voor de patiënt van toegevoegde waarde. | All things considered, I think the deferred consent procedure adds value to the patient. |
| Alles samengenomen vind ik de deferred consent procedure voor het onderzoek van toegevoegde waarde. | All things considered, I think the deferred consent procedure adds value to the study. |

**Theme 4.** Prescribing of randomized empirical treatment

| Dutch | English |
| --- | --- |
| Het ziekenhuispersoneel (bv. artsen en verpleegkundigen) had bezwaar tegen het volgen van de gerandomiseerde behandeling. | Clinical staff (e.g., physicians and nurses) objected to adhering to the randomized treatment. |
| Het ziekenhuispersoneel (bv. artsen en verpleegkundigen) dacht dat de patiënten minder goed behandeld zouden worden door de gerandomiseerde behandeling. | Clinical staff (e.g., physicians and nurses) thought that patients would receive inferior treatment with the randomized treatment. |
| De angst voor mogelijke bijwerkingen belemmerde het voorschrijven van de gerandomiseerde behandeling. | Fear of potential side effects hindered the prescribing of the randomized treatment. |
| De toedieningsfrequentie van de gerandomiseerde behandeling belemmerde de artsen en verpleegkundigen om de gerandomiseerde behandeling voor te schrijven dan wel toe te dienen. | The frequency of administration of the randomized treatment hindered physicians and nurses from prescribing or administering the randomized treatment. |

**Barrieres to prescribing the randomized empirical treatment**

| Dutch | English |
| --- | --- |
| Onbekendheid met het lokale antibioticaprotocol/de gerandomiseerde behandeling | Unfamiliarity with the local antibiotic protocol / the randomized treatment. |
| Onvoldoende overtuigd van een hogere effectiviteit van de gerandomiseerde behandeling | Insufficiently convinced of the higher effectiveness of the randomized treatment. |
| Mindere effectiviteit van de gerandomiseerde behandeling | Lower effectiveness of the randomized treatment. |
| Toedieningsfrequentie | Frequency of administration. |
| Angst voor mogelijke bijwerkingen | Concerns for potential side effects. |
| Overig | Other. |

## S2. Translation of the questionnaire – Clinical staff

**Theme 5.** Prescribing of randomized empirical treatment

| Dutch | English |
| --- | --- |
| Ik had bezwaar tegen het volgen van de gerandomiseerde behandeling. | I objected to adhering to the randomized treatment. |
| Ik dacht dat de patiënten minder goed behandeld zouden worden door de gerandomiseerde behandeling. | I thought that patients would receive inferior treatment with the randomized treatment. |
| De angst voor mogelijke bijwerkingen belemmerde mij in het voorschrijven van de gerandomiseerde behandeling. | The fear of potential side effects hindered me from prescribing the randomized treatment. |
| De toedieningsfrequentie van de gerandomiseerde behandeling belemmerde mij om de gerandomiseerde behandeling voor te schrijven dan wel toe te dienen. | The frequency of administration of the randomized treatment hindered me from prescribing or administering the randomized treatment. |
| Ik heb een ervaring met het geven van monotherapie, die mij terughoudend maakt om monotherapie te geven. | I have an experience with administering monotherapy, which makes me hesitant to prescribe it. |
| Ik heb een ervaring met het geven van combinatietherapie, die mij terughoudend maakt om combinatietherapie te geven. | I have an experience with administering combination therapy, which makes me hesitant to prescribe it. |
| Bij het voorschrijven van de antibioticatherapie, heeft mijn arts/supervisor wel eens geadviseerd af te wijken van de gerandomiseerde behandeling. | When prescribing antibiotic treatment, my physician / supervisor has, at times, advised deviation from the randomized treatment. |

**Barrieres to prescribing the randomized empirical treatment**

| Dutch | English |
| --- | --- |
| Onbekendheid met het lokale antibioticaprotocol/de gerandomiseerde behandeling | Unfamiliarity with the local antibiotic protocol / the randomized treatment. |
| Onvoldoende overtuigd van een hogere effectiviteit van de gerandomiseerde behandeling | Insufficiently convinced of the higher effectiveness of the randomized treatment. |
| Mindere effectiviteit van de gerandomiseerde behandeling | Lower effectiveness of the randomized treatment. |
| Arts/supervisor die adviseert af te wijken | Physician / supervisor recommending deviation. |
| Toedieningsfrequentie | Frequency of administration. |
| Angst voor mogelijke bijwerkingen | Concerns for potential side effects. |
| Overig | Other. |

## S3. Explanation and practical example of relative trial efficiency calculation

The relative trial efficiency reflects the relative decrease in duration of the trial enrolment period after adding an exclusion criterion (alternative scenario) compared to the actual trial (original scenario). We are assuming that the compliance in the two scenarios is known and taken into account in the sample size calculation and are further assuming that the enrolment capacity per site per year remains the same. Its calculation includes two components:

1. **Taking into account the compliance in the sample size calculation (“compliance effect”):**

Non-compliance in the original scenario is 46% in the combination arm and 9% in the monotherapy arm, as in the SAGA trial. This inflates the required sample size by a factor:

$$I= \frac{1}{\left( 1-e1-e2 \right)^2}$$

Where e1 and e2 are the non-compliance rates in the two arms. For the given scenario, this would be 1 / (1-0.46-0.09)^2^ = 4.94.

When excluding patients with kidney function at presentation < 30 ml/min, non-compliance in the combination therapy arm would have decreased from 0.46 to 0.43. The inflation fraction would have reduced to 1 / (1-0.43-0.09)^2^ = 4.34. The compliance effect would then be 4.94 / 4.34 = 1.14 under this alternative scenario. (Note that being 14% more efficient with the slightly higher compliance means requiring 12% fewer patients in the trial.)

1. **Discounting that fewer patients are eligible for the trial (“Eligibility effect”)**

In the alternative scenario, the proportion of patients eligible under the stricter criteria decreased from 263 to 235, representing 0.89 of the original sample (i.e. the prevalence of kidney function at presentation < 30 ml/min is 11%). Note that having 11% fewer eligible patients normally means that the trial duration is increased by a factor 1.12.

**Relative trial efficiency**

The relative trial efficiency is defined as the product of the compliance effect and the eligibility effect. In the given example, trial efficiency is 1.02. To conclude, excluding patients with a kidney function < 30 ml/min at admission would have resulted in a 2% increase in trial efficiency. This means that we could have finalized the trial slightly earlier by excluding these patients, although the gain is too small to be meaningful.

For all other tested scenarios, excluding patients with higher non-compliance resulted in a relative trial efficiency <1. The trial would only last longer in these cases.

**Scenario resulting in relative trial efficiency gain >1**

Suppose that a subgroup X, with a prevalence of 30% of the original population has a very high non-compliance in the combination therapy arm (83%) while it is indifferent for monotherapy (9% non-compliance). Excluding this population would decrease the compliance in the combination therapy arm to 30%. The resulting inflation factor would be 1 / (1-0.30-0.09)^2^ = 2.69, resulting in a compliance effect of 4.93/2.69 = 1.84.

The eligibility effect would be 1-prevalence = 0.70. The relative trial efficiency is 1.29. In this hypothetical situation, we would yield an approximately 22% shorter enrolment time.

## S4. Results of training and preparation

*
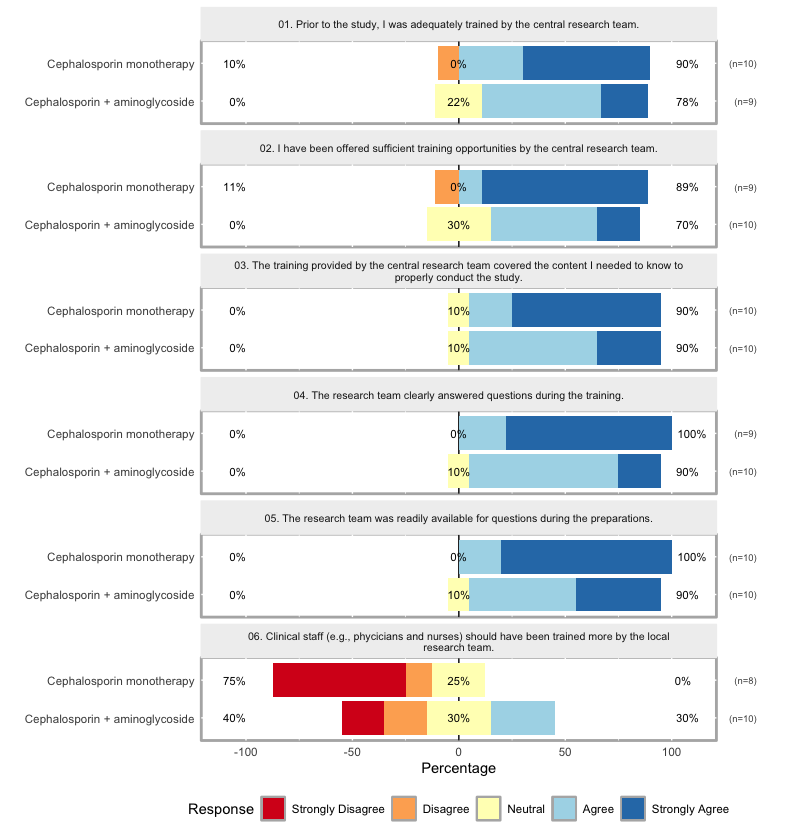
*

*Figure S4. Training and preparation*

## S5. Results of study procedures


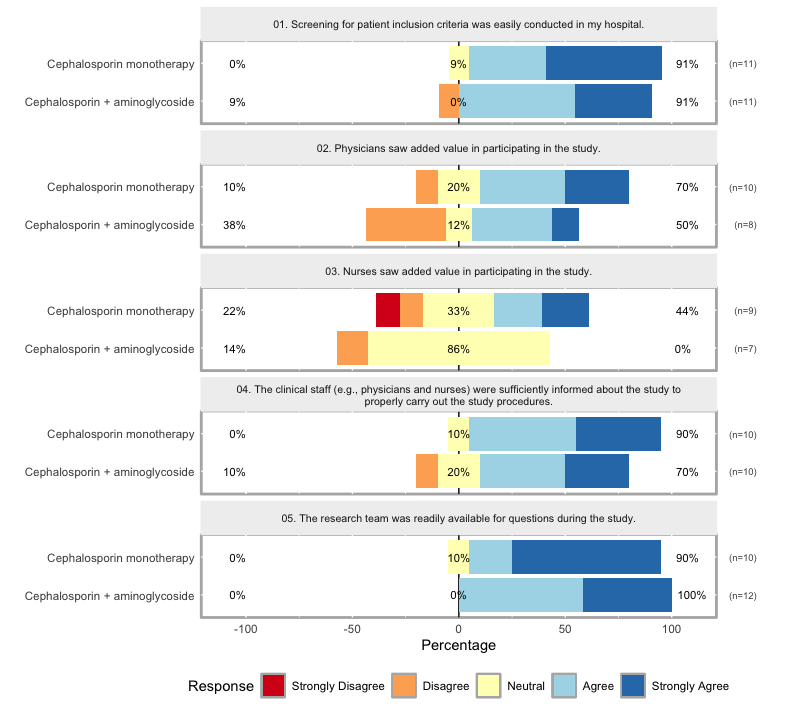


*Figure S5. Study procedures*

## S6. Results of deferred consent procedure


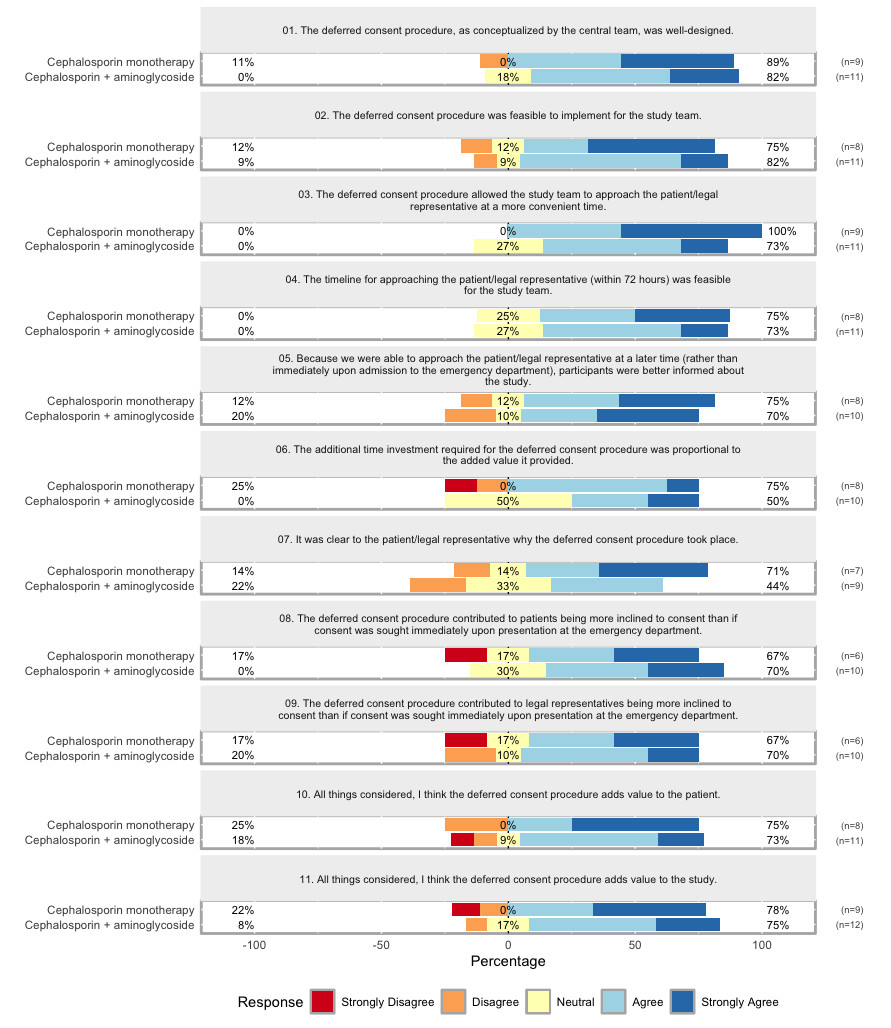


*Figure S6. Deferred consent procedure*

## S7. Results of prescribing randomized empirical treatment – Local research teams


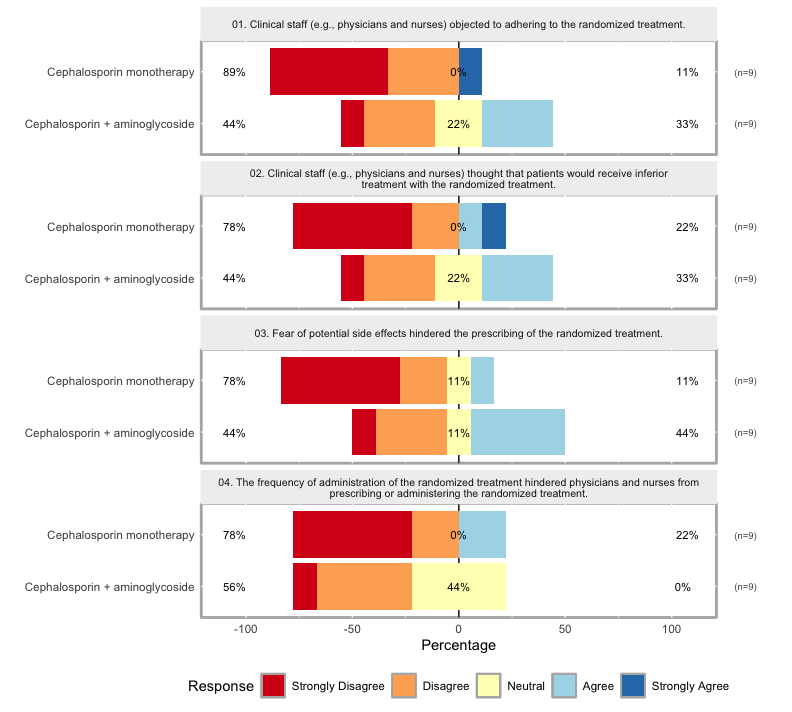


*Figure S7. Prescribing of randomized empirical treatment*

## S8. Results of prescribing randomized empirical treatment – Physicians

*
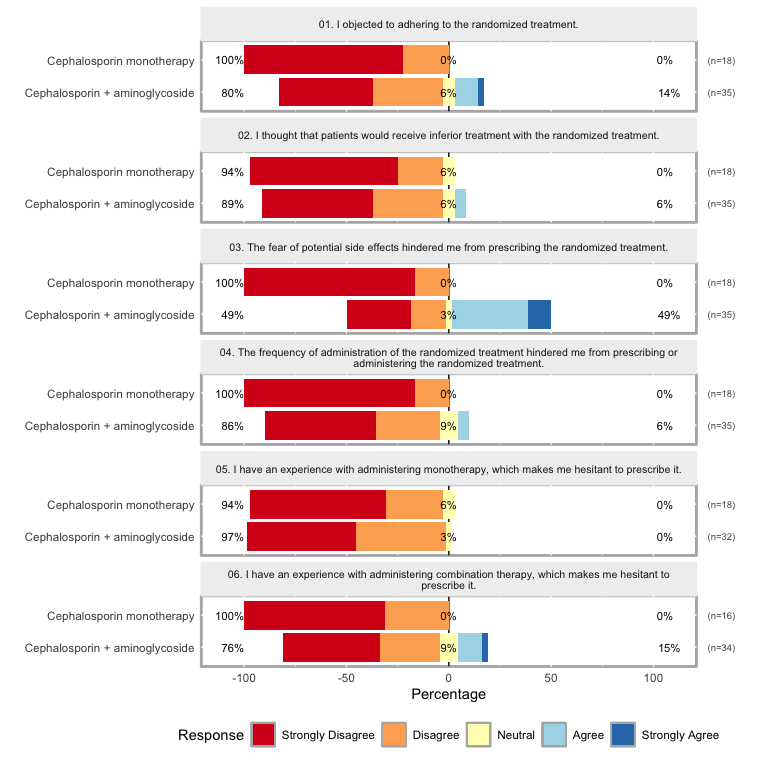
*

*Figure S8. Prescribing of randomized empirical treatment*

*
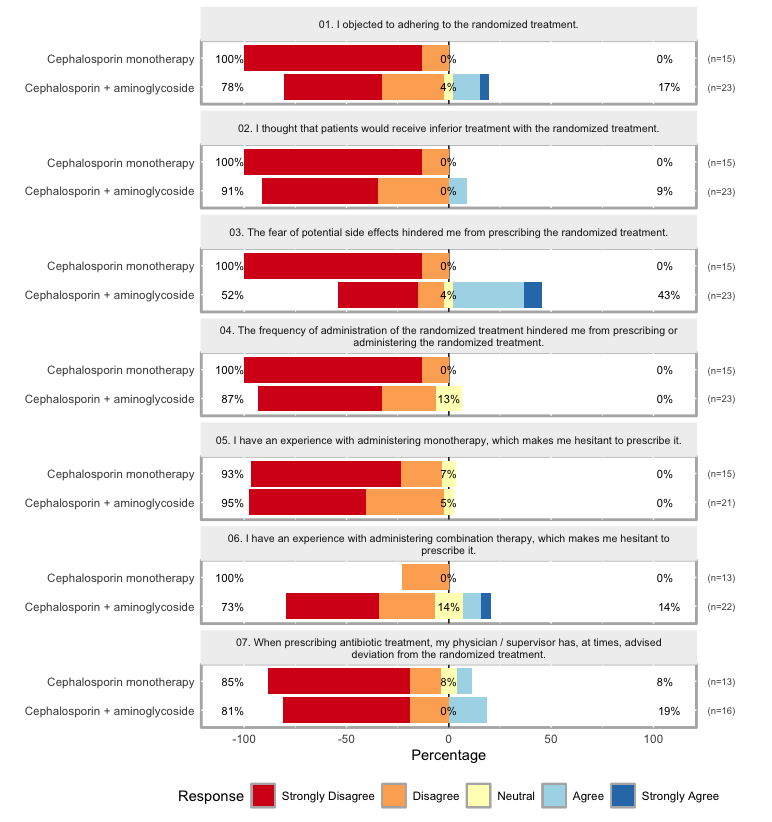
*

*Figure S8a. Prescribing of randomized empirical treatment – Medical specialists*

*
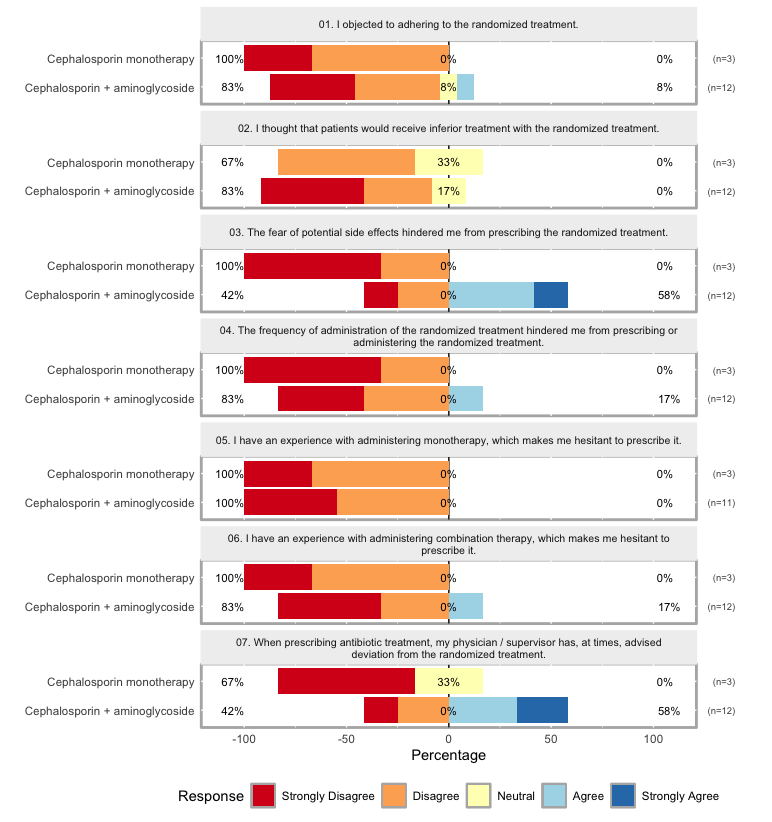
*

*Figure S8b. Prescribing of randomized empirical treatment - Residents*
