# Supplementary material for: Failure to rescue the trial: lessons from a randomised antibiotic treatment trial in the acute care setting
Source: Trials. 2026 Apr 23;27:418. doi: 10.1186/s13063-026-09722-3 (PMC13237994; doi:10.1186/s13063-026-09722-3)
Supplement: Supplementary file 2 — Supplementary Material 2. [file 13063_2026_9722_MOESM2_ESM.docx]

# Reporting checklist for randomised trial.

Based on the CONSORT guidelines.

## Instructions to authors

Complete this checklist by entering the page numbers from your manuscript where readers will find each of the items listed below.

Your article may not currently address all the items on the checklist. Please modify your text to include the missing information. If you are certain that an item does not apply, please write "n/a" and provide a short explanation.

Upload your completed checklist as an extra file when you submit to a journal.

In your methods section, say that you used the CONSORTreporting guidelines, and cite them as:

Schulz KF, Altman DG, Moher D, for the CONSORT Group. CONSORT 2010 Statement: updated guidelines for reporting parallel group randomised trials

|  |  | Reporting Item | Page Number |
| --- | --- | --- | --- |
| **Title and Abstract** |  |  |  |
| Title | [#1a](https://www.goodreports.org/reporting-checklists/consort/info/#1a) | Identification as a randomized trial in the title. | 1 |
| Abstract | [#1b](https://www.goodreports.org/reporting-checklists/consort/info/#1b) | Structured summary of trial design, methods, results, and conclusions | 2 |
| **Introduction** |  |  |  |
| Background and objectives | [#2a](https://www.goodreports.org/reporting-checklists/consort/info/#2a) | Scientific background and explanation of rationale | 3 |
| Background and objectives | [#2b](https://www.goodreports.org/reporting-checklists/consort/info/#2b) | Specific objectives or hypothesis | 3 |
| **Methods** |  |  |  |
| Trial design | [#3a](https://www.goodreports.org/reporting-checklists/consort/info/#3a) | Description of trial design (such as parallel, factorial) including allocation ratio. | 3: current study and article “Failure to rescue the trial: lessons from a randomised antibiotic treatment trial in the acute care setting” (https://doi.org/10.1016/j.cmicom.2025.105075): for the cluster-randomized cross-over trial |
| Trial design | [#3b](https://www.goodreports.org/reporting-checklists/consort/info/#3b) | Important changes to methods after trial commencement (such as eligibility criteria), with reasons | https://doi.org/10.1016/j.cmicom.2025.105075: for the cluster-randomized cross-over trial |
| Participants | [#4a](https://www.goodreports.org/reporting-checklists/consort/info/#4a) | Eligibility criteria for participants | 4: current study and https://doi.org/10.1016/j.cmicom.2025.105075: for the cluster-randomized cross-over trial |
| Participants | [#4b](https://www.goodreports.org/reporting-checklists/consort/info/#4b) | Settings and locations where the data were collected | 4: current study and https://doi.org/10.1016/j.cmicom.2025.105075: for the cluster-randomized cross-over trial |
| Interventions | [#5](https://www.goodreports.org/reporting-checklists/consort/info/#5) | The experimental and control interventions for each group with sufficient details to allow replication, including how and when they were actually administered | https://doi.org/10.1016/j.cmicom.2025.105075: for the cluster-randomized cross-over trial |
| Outcomes | [#6a](https://www.goodreports.org/reporting-checklists/consort/info/#6a) | Completely defined prespecified primary and secondary outcome measures, including how and when they were assessed | 4, https://doi.org/10.1016/j.cmicom.2025.105075: for the cluster-randomized cross-over trial |
| Sample size | [#7a](https://www.goodreports.org/reporting-checklists/consort/info/#7a) | How sample size was determined. | N/A: current study and https://doi.org/10.1016/j.cmicom.2025.105075: for the cluster-randomized cross-over trial |
| Sample size | [#7b](https://www.goodreports.org/reporting-checklists/consort/info/#7b) | When applicable, explanation of any interim analyses and stopping guidelines | N/A |
| Randomization - Sequence generation | [#8a](https://www.goodreports.org/reporting-checklists/consort/info/#8a) | Method used to generate the random allocation sequence. |  |
| https://doi.org/10.1016/j.cmicom.2025.105075: for the cluster-randomized cross-over trial |  |  |  |
| Randomization - Sequence generation | [#8b](https://www.goodreports.org/reporting-checklists/consort/info/#8b) | Type of randomization; details of any restriction (such as blocking and block size) |  |
| https://doi.org/10.1016/j.cmicom.2025.105075: for the cluster-randomized cross-over trial |  |  |  |
| Randomization - Allocation concealment mechanism | [#9](https://www.goodreports.org/reporting-checklists/consort/info/#9) | Mechanism used to implement the random allocation sequence (such as sequentially numbered containers), describing any steps taken to conceal the sequence until interventions were assigned | N/A, cluster-randomized cross-over trial |
| Randomization - Implementation | [#10](https://www.goodreports.org/reporting-checklists/consort/info/#10) | Who generated the allocation sequence, who enrolled participants, and who assigned participants to interventions | https://doi.org/10.1016/j.cmicom.2025.105075: for the cluster-randomized cross-over trial |
| Blinding | [#11a](https://www.goodreports.org/reporting-checklists/consort/info/#11a) | If done, who was blinded after assignment to interventions (for example, participants, care providers, those assessing outcomes) and how. | N/A, not blinded |
| Blinding | [#11b](https://www.goodreports.org/reporting-checklists/consort/info/#11b) | If relevant, description of the similarity of interventions | N/A, not blinded |
| Statistical methods | [#12a](https://www.goodreports.org/reporting-checklists/consort/info/#12a) | Statistical methods used to compare groups for primary and secondary outcomes | https://doi.org/10.1016/j.cmicom.2025.105075: for the cluster-randomized cross-over trial |
| Statistical methods | [#12b](https://www.goodreports.org/reporting-checklists/consort/info/#12b) | Methods for additional analyses, such as subgroup analyses and adjusted analyses | 4, 5: current study https://doi.org/10.1016/j.cmicom.2025.105075: for the cluster-randomized cross-over trial |
| Outcomes | [#6b](https://www.goodreports.org/reporting-checklists/consort/info/#6b) | Any changes to trial outcomes after the trial commenced, with reasons | N/A, no changes to trial outcomes |
| **Results** |  |  |  |
| Participant flow diagram (strongly recommended) | [#13a](https://www.goodreports.org/reporting-checklists/consort/info/#13a) | For each group, the numbers of participants who were randomly assigned, received intended treatment, and were analysed for the primary outcome | https://doi.org/10.1016/j.cmicom.2025.105075: for the cluster-randomized cross-over trial |
| Participant flow | [#13b](https://www.goodreports.org/reporting-checklists/consort/info/#13b) | For each group, losses and exclusions after randomization, together with reason | https://doi.org/10.1016/j.cmicom.2025.105075: for the cluster-randomized cross-over trial |
| Recruitment | [#14a](https://www.goodreports.org/reporting-checklists/consort/info/#14a) | Dates defining the periods of recruitment and follow-up | 4, and https://doi.org/10.1016/j.cmicom.2025.105075: for the cluster-randomized cross-over trial |
| Recruitment | [#14b](https://www.goodreports.org/reporting-checklists/consort/info/#14b) | Why the trial ended or was stopped | 1, and https://doi.org/10.1016/j.cmicom.2025.105075: for the cluster-randomized cross-over trial |
| Baseline data | [#15](https://www.goodreports.org/reporting-checklists/consort/info/#15) | A table showing baseline demographic and clinical characteristics for each group | 13: current study and https://doi.org/10.1016/j.cmicom.2025.105075: for the cluster-randomized cross-over trial |
| Numbers analysed | [#16](https://www.goodreports.org/reporting-checklists/consort/info/#16) | For each group, number of participants (denominator) included in each analysis and whether the analysis was by original assigned groups | 14: current study and https://doi.org/10.1016/j.cmicom.2025.105075: for the cluster-randomized cross-over trial |
| Outcomes and estimation | [#17a](https://www.goodreports.org/reporting-checklists/consort/info/#17a) | For each primary and secondary outcome, results for each group, and the estimated effect size and its precision (such as 95% confidence interval) | 14: current study and https://doi.org/10.1016/j.cmicom.2025.105075: for the cluster-randomized cross-over trial |
| Outcomes and estimation | [#17b](https://www.goodreports.org/reporting-checklists/consort/info/#17b) | For binary outcomes, presentation of both absolute and relative effect sizes is recommended | https://doi.org/10.1016/j.cmicom.2025.105075 |
| Ancillary analyses | [#18](https://www.goodreports.org/reporting-checklists/consort/info/#18) | Results of any other analyses performed, including subgroup analyses and adjusted analyses, distinguishing pre-specified from exploratory | https://doi.org/10.1016/j.cmicom.2025.105075 |
| Harms | [#19](https://www.goodreports.org/reporting-checklists/consort/info/#19) | All important harms or unintended effects in each group (For specific guidance see CONSORT for harms) | https://doi.org/10.1016/j.cmicom.2025.105075 |
| **Discussion** |  |  |  |
| Limitations | [#20](https://www.goodreports.org/reporting-checklists/consort/info/#20) | Trial limitations, addressing sources of potential bias, imprecision, and, if relevant, multiplicity of analyses | 9 and https://doi.org/10.1016/j.cmicom.2025.105075 |
| Interpretation | [#22](https://www.goodreports.org/reporting-checklists/consort/info/#22) | Interpretation consistent with results, balancing benefits and harms, and considering other relevant evidence | https://doi.org/10.1016/j.cmicom.2025.105075 |
| Registration | [#23](https://www.goodreports.org/reporting-checklists/consort/info/#23) | Registration number and name of trial registry | 2 |
| Generalisability | [#21](https://www.goodreports.org/reporting-checklists/consort/info/#21) | Generalisability (external validity, applicability) of the trial findings | https://doi.org/10.1016/j.cmicom.2025.105075 |
| **Other information** |  |  |  |
| Interpretation | [#22](https://www.goodreports.org/reporting-checklists/consort/info/#22) | Interpretation consistent with results, balancing benefits and harms, and considering other relevant evidence | https://doi.org/10.1016/j.cmicom.2025.105075 |
| Registration | [#23](https://www.goodreports.org/reporting-checklists/consort/info/#23) | Registration number and name of trial registry | 2 |
| Protocol | [#24](https://www.goodreports.org/reporting-checklists/consort/info/#24) | Where the full trial protocol can be accessed, if available | N/A |
| Funding | [#25](https://www.goodreports.org/reporting-checklists/consort/info/#25) | Sources of funding and other support (such as supply of drugs), role of funders | 10: for current study, and https://doi.org/10.1016/j.cmicom.2025.105075: RCT |

Notes:

- 3a: 3: current study and article “Failure to rescue the trial: lessons from a randomised antibiotic treatment trial in the acute care setting” (https://doi.org/10.1016/j.cmicom.2025.105075): for the cluster-randomized cross-over trial
- 4a: 4: current study and https://doi.org/10.1016/j.cmicom.2025.105075: for the cluster-randomized cross-over trial
- 4b: 4: current study and https://doi.org/10.1016/j.cmicom.2025.105075: for the cluster-randomized cross-over trial
- 5: https://doi.org/10.1016/j.cmicom.2025.105075: for the cluster-randomized cross-over trial
- 6a: 4, https://doi.org/10.1016/j.cmicom.2025.105075: for the cluster-randomized cross-over trial
- 7a: N/A: current study and https://doi.org/10.1016/j.cmicom.2025.105075: for the cluster-randomized cross-over trial
- 8a: https://doi.org/10.1016/j.cmicom.2025.105075: for the cluster-randomized cross-over trial
- 8b: N/A: current study and https://doi.org/10.1016/j.cmicom.2025.105075: for the cluster-randomized cross-over trial
- 9: N/A, cluster-randomized cross-over trial
- 10: https://doi.org/10.1016/j.cmicom.2025.105075: for the cluster-randomized cross-over trial
- 11a: N/A, not blinded
- 11b: N/A, not blinded
- 12a: https://doi.org/10.1016/j.cmicom.2025.105075: for the cluster-randomized cross-over trial
- 12b: 4,5: current study and https://doi.org/10.1016/j.cmicom.2025.105075: for the cluster-randomized cross-over trial
- 13a: https://doi.org/10.1016/j.cmicom.2025.105075: for the cluster-randomized cross-over trial
- 13b: https://doi.org/10.1016/j.cmicom.2025.105075: for the cluster-randomized cross-over trial
- 14a: 4, and https://doi.org/10.1016/j.cmicom.2025.105075: for the cluster-randomized cross-over trial
- 14b: 1, and https://doi.org/10.1016/j.cmicom.2025.105075: for the cluster-randomized cross-over trial
- 15: 13 and https://doi.org/10.1016/j.cmicom.2025.105075: for the cluster-randomized cross-over trial
- 16: 14 and https://doi.org/10.1016/j.cmicom.2025.105075: for the cluster-randomized cross-over trial
- 17a: 14 and https://doi.org/10.1016/j.cmicom.2025.105075: for the cluster-randomized cross-over trial
- 17b: https://doi.org/10.1016/j.cmicom.2025.105075: for the cluster-randomized cross-over trial
- 18: https://doi.org/10.1016/j.cmicom.2025.105075: for the cluster-randomized cross-over trial
- 19: https://doi.org/10.1016/j.cmicom.2025.105075: for the cluster-randomized cross-over trial
- 20: 9 and https://doi.org/10.1016/j.cmicom.2025.105075: for the cluster-randomized cross-over trial
- 21: https://doi.org/10.1016/j.cmicom.2025.105075: for the cluster-randomized cross-over trial
- 22: https://doi.org/10.1016/j.cmicom.2025.105075: for the cluster-randomized cross-over trial
- 25: 10: for current study, and https://doi.org/10.1016/j.cmicom.2025.105075: for the cluster-randomized cross-over trial
- The CONSORT checklist is distributed under the terms of the Creative Commons Attribution License CC-BY. This checklist was completed on 17. September 2025 using <https://www.goodreports.org/>, a tool made by the [EQUATOR Network](https://www.equator-network.org) in collaboration with [Penelope.ai](https://www.penelope.ai)
